# Real-time monitoring of mono- and dual-species biofilm formation and eradication using microfluidic platform

**DOI:** 10.1038/s41598-022-13699-9

**Published:** 2022-06-11

**Authors:** Van Nam Tran, Fazlurrahman Khan, Won Han, Maknuna Luluil, Van Gia Truong, Hyo Geun Yun, Sungyoung Choi, Young-Mog Kim, Joong Ho Shin, Hyun Wook Kang

**Affiliations:** 1 Industry 4.0 Convergence Bionics Engineering and Marine-Integrated Biomedical Technology Center, Pukyong National University, Busan, 48513 South Korea; 2 Research Center for Marine Integrated Bionics Technology, Pukyong National University, Busan, 48513 South Korea; 3 Department of Biomedical Engineering, Pukyong National University, Busan, 48513 South Korea; 4 Department of Electronic Engineering, Hanyang University, Seoul, 04763 South Korea; 5 Department of Biomedical Engineering, Hanyang University, Seoul, 04763 South Korea; 6 Department of Food Science and Technology, Pukyong National University, Busan, 48513 South Korea

**Keywords:** Biological techniques, Microbiology, Molecular biology, Nanoscience and technology, Optics and photonics

## Abstract

In a human host, bacterial *Staphylococcus aureus* and fungal *Candida albicans* pathogens form a mixed biofilm that causes severe mortality and morbidity. However, research on the formation and eradication of mixed biofilms under dynamic conditions is lacking. Thus, this study employed a microfluidic technique to analyze the real-time formation of mono- and dual-species (*S. aureus* and *C. albicans*) biofilms and noninvasive optical treatment of the established mature biofilm using 405-nm laser light. A herringbone mixer thoroughly mixed both bacterial and fungal cells in the growth media before being injected into the observation channels on the microfluidic chip. At a flow rate of 1.0 µL/min of growth media for 24 h, the bacterial biofilm coverage was up to 15% higher than that of the fungal biofilm (50% for bacteria vs. 35% for fungus). On the other hand, the dual-species biofilm yielded the highest coverage of ~ 96.5% because of the collective interaction between *S. aureus* and *C. albicans*. The number of cell proliferation events in *S. aureus* was higher than that of *C. albicans* for 12 h, which indicates that the *S. aureus* biofilm was developed faster than *C. albicans*. The novel in situ test platform showed a significant bactericidal effect (80%) of the 405-nm laser light at 1080 J/cm^2^ towards the established *S. aureus* biofilm, whereas the same treatment removed approximately 69% of the mixed cells in the dual-species biofilm. This study revealed that the developed microfluidic platform could be utilized to monitor the formation of dual-species biofilms in real-time and laser-induced antimicrobial effects on dual-species biofilms.

## Introduction

Microorganisms exist in complex communities, including host, clinical, and industrial environments^1,2^. The presence of complex microbial communities is beneficial to either single or both microbial species in the environment^3^. Furthermore, polymicrobial interactions have numerous advantages, including changes in host immune responses, microbial pathogenesis, and virulence^4^. Polymicrobial interactions in numerous natural environments, including the host, result in the formation of biofilms, which contain heterogeneous microcolonies of microbial cells surrounded by self-produced extracellular polymeric substances^5^. The microbial populations in the biofilm largely benefit each other in terms of exchange of nutrients, metabolites, signalling molecules, genetic materials, resistance to antimicrobial treatment, and protection from host immunological responses^6–8^.

Infections caused by polymicrobial biofilms resulted in a higher mortality rate (70%) than that caused by mono-species biofilms^9^. The majority of information on the process of biofilm formation and virulence characteristics has been gathered from a single bacterial species^9^. Most of the susceptibility testing for antimicrobial agents is entirely based on mono-species planktonic cell culture, which increases the chance of failure to treat polymicrobial infections^10,11^. In the contemporary context, research on polymicrobial biofilms employing multi-species microbial communities is of considerable interest in determining the true impact of the polymicrobial biofilm on the natural host, clinical, and industrial settings^4^. Researchers are focused on the interaction between fungi and bacteria to understand polymicrobial interactions at a cross-kingdom level^12,13^. Fungal pathogens, particularly *Candida albicans*, have frequently been co-isolated with numerous pathogenic bacterial species from the oral cavity, skin and mucosal surface, gastrointestinal tract, lungs, and vulvovaginal surface^14,15^. *Staphylococcus aureus* and *C. albicans* have been identified together from various biofilm-associated illnesses, including cystic fibrosis, keratitis, ventilator-associated pneumonia, denture stomatitis, periodontitis, urinary tract, and burn wound infections, as reviewed by Carolus et al.^14^. *S. aureus* produces several virulence factors, including proteases, hemolysin, leukocidins, enterotoxin, immune-modulatory molecules, and exfoliating toxin^16^. Similarly, the *C. albicans* pathogenicity exhibited various virulence factors, such as morphological changes, biofilm development, adhesin production, hydrolytic enzyme release, and penetration of the host cell surface^4,17^.

Several investigations have shown that the interaction between *S. aureus* and *C. albicans* involved both physical and chemical interactions^14,18^. The hyphae-specific receptor protein (Als3p) is involved in the physical interaction between *S. aureus* and *C. albicans*, whereas quorum sensing (QS) siganlling molecules are involved in the chemical interaction^15^. Such interactions lead to an enhanced biofilm formation and development of resistance mechanisms against antimicrobial agents^19^. Furthermore, the coinfection of *S. aureus* and *C. albicans* to the animal model organism resulted in an increased microbial load in the host organs as well as a significant mortality rate^20,21^. In polymicrobial infections, *S. aureus* and *C. albicans* share a common niche in the human body, such as the skin, vagina, nasal passage, pharynx, abdominal cavity, and oral mucosa^22^. Physical interactions are established by *S. aureus* adhering to the cell surface of *C. albicans*, whereas the chemical interaction is mediated by the participation of QS signalling molecules, secretory chemicals generated either by *S. aureus* and *C. albicans*, or human host^14,23^. Despite the success of the in vitro research on elucidating the physical interplay between *S. aureus* and *C. albicans*, in vivo studies are necessary to understand the interaction mechanism in various host tissues and organs, which are the primary cause of synergistic lethality. Furthermore, the flow of bodily fluids and secretions exposes microbial pathogens, including *S. aureus* and *C. albicans*, in numerous human and animal hosts body areas, such as the mouth, lung, intestine, stomach, and urinary system^24^. The majority of in vitro polymicrobial interactions and formation of dual-species biofilms between *S. aureus* and *C. albicans*, have been investigated under static conditions. However, research on the formation of polymicrobial biofilms under dynamic conditions is lacking.

Recently, integrated microfluidics was identified as a promising approach to study microbial interactions^25^. Several other important phenomena have also been investigated, including the influences of physical and chemical factors on cell viability, proliferation, functionality, and motility^10,26^. Furthermore, the microfluidic system can help understand the effects of hydrodynamic factors, such as flow rate and shear forces, on the initial stage of biofilm development and cell morphology^26^. Crabbé et al. agreed that shear forces affect microbial adherence, persistence, and hence infection capacity of opportunistic pathogens^27^. Considering this, several reports have demonstrated the use of microfluidics to study biofilm formation at a single species level, to test the influence of hydrodynamic conditions, bacterial chemotaxis, and screening of antibiotics or antibiofilm drugs^26,28^. Similarly, other studies investigated the real-time monitoring of biofilm formation at the single species level^29–32^. Furthermore, the microfluidic system has been used to study host–pathogen interactions^33^. Although multi-species biofilm formation has been performed using a microfluidic device^34^, such as in dental plaque, relatively few studies have investigated real-time monitoring of biofilm production at the multi-species level^35,36^. The implementation of the microfluidic method can aid in monitoring the mono- and dual-species biofilm formation between *S. aureus* and *C. albicans* in real-time. Besides, an optical treatment for the bacterial biofilms using 405-nm laser light has been widely deployed to evaluate the antibacterial effect on bacterial biofilms in vitro^37^; however, the research on the 405-nm laser light for dual-species biofilm eradication with the assistance of the microfluidic platform under hydrodynamic conditions is lacking. Therefore, the current study intended to develop a versatile, yet simple microfluidic platform to investigate the formation of mono- and dual-species biofilms of *S. aureus* and *C. albicans* in real-time. Furthermore, a physical approach, photoirradiation with 405-nm laser light, was used to eradicate the developed mature mono- and dual-species biofilms of *S. aureus* and *C. albicans* under hydrodynamic circumstances.

## Results

### Passing of media and cell culture into the channel

**Figure 1:**
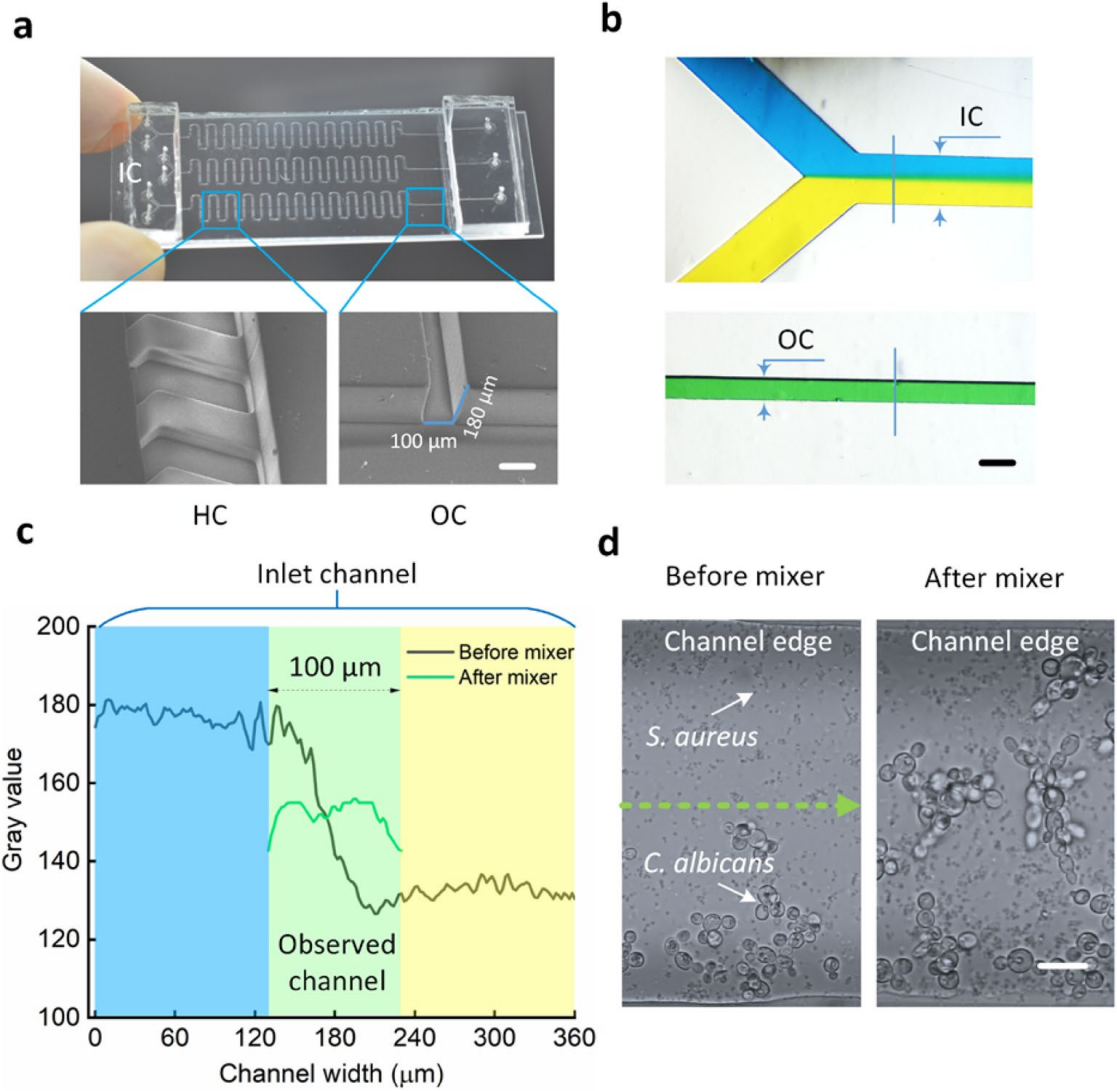
Microfluidic channel visualization and mixing capability: (**a**) overview of the proposed microfluidic chip (top) and scanning electron microscopy (SEM) images of herringbone (bottom-left) and observed (bottom-right) channels (*IC* inlet channel, *HC* herringbone channel, *OC* observed channel; scale bar in (**a**) = 100 µm), (**b**) colour mixing effect before (top) and after (bottom) the herringbone mixer (scale bar in (**b**) = 100 µm), (**c**) grey value measured from blue lines on colour mixing images (**b**) before and after the herringbone mixer, and (**d**) bacterial and fungal mixtures before and after the herringbone mixer at the injection phase (scale bar in (**d**) = 10 µm). The top line represents inlet channels with two apparent colour areas (blue and yellow), which were then mixed thoroughly into a green colour area on the observed channel.

Figure 1 shows images of the designed chip as well as the correlation between the colour mixing effect and biofilm formation generated by the herringbone mixer. The microfluidic channel was designed to be 100 µm wide and 180 µm tall (Fig. 1a). As a result, the chip allowed researchers to investigate adhesion, proliferation, and diffusion at the single-cell level during biofilm development. The mixing effect of the two colours (yellow and blue) before and after the herringbone mixer is depicted in Fig. 1b. Prior to the mixing, two-colour regions were visible (top), while the green-like colour resulted from the good mixing (bottom). This effect is shown in Fig. 1c. In the case of nonmixing deployment, the grey value and channel width changed significantly. In contrast, the grey value became almost constant across the investigated breadth of the observed channel. As a result, during the injection phase, the mixing capability of the proposed chip with two microorganisms was clearly distinguished on the inlet and observed channels. Two apparent areas of bacteria and fungi were identified in the inlet channel. Notably, a mixed culture was observed after the herringbone mixing (Fig. 1d).

### Optical setup optimization

**Figure 2:**
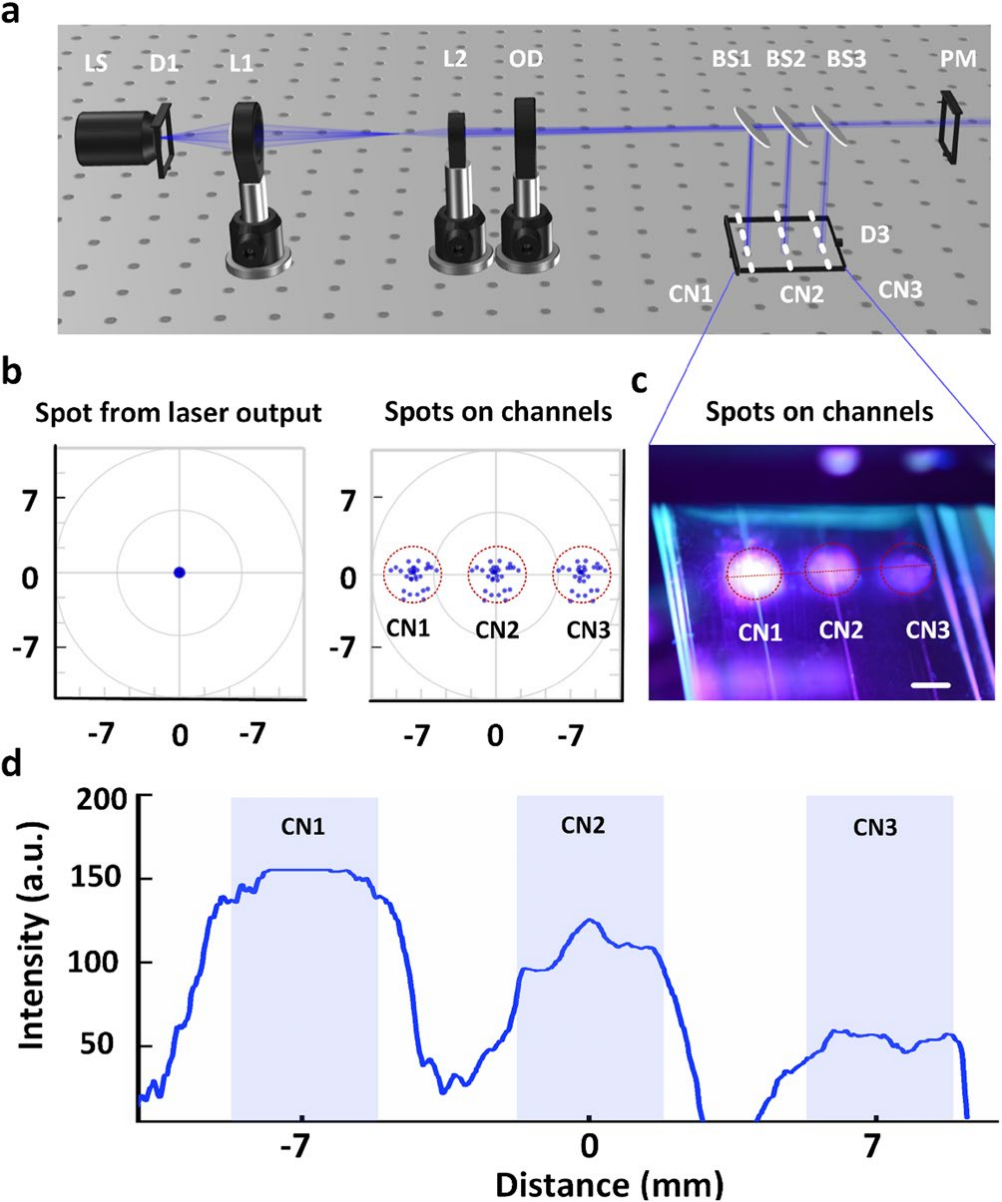
Optical system setup for biofilm treatment: (**a**) optical model setup for simulation, (**b**) simulated beam spots emitted from the laser output (left) and on three observed channels (right), (**c**) measured laser beam spots on the three observed channels in the proposed microfluidic chip, and (**d**) light intensity quantified from the three laser beam spots on the three observed channels (*LS* laser source, *D1 and D2* optical detectors, *L1 and L2* lenses, *OD* optical diffuser, *BS1, BS2, and BS3* beam splitters, *PM* power meter, *CN1, CN2, and CN3* observed channels from 1 to 3 on the microfluidic chip; scale bar = 3 mm).

Figure 2a shows an optical simulation of a laser beam propagating through an optical system. The diverging output beam (Gaussian distribution and numerical aperture = 0.22) from the laser system was uniformly collimated and delivered to three channels with equal beam spots of 5 mm (Fig. 2b; left and middle). The three power levels on these channels were 50, 25, and 12.5% of the output power from the laser system, respectively. The experimental results are shown in Fig. 2c to validate the simulation. The light emissions of the three flat-top beams on the chip surface indicated that the Gaussian beam from the laser source diffused into uniform spots on the channel surface. The intensities were 50, 100, and 150 for each unit, respectively (Fig. 2d).

### Mono-species biofilm formation

**Figure 3:**
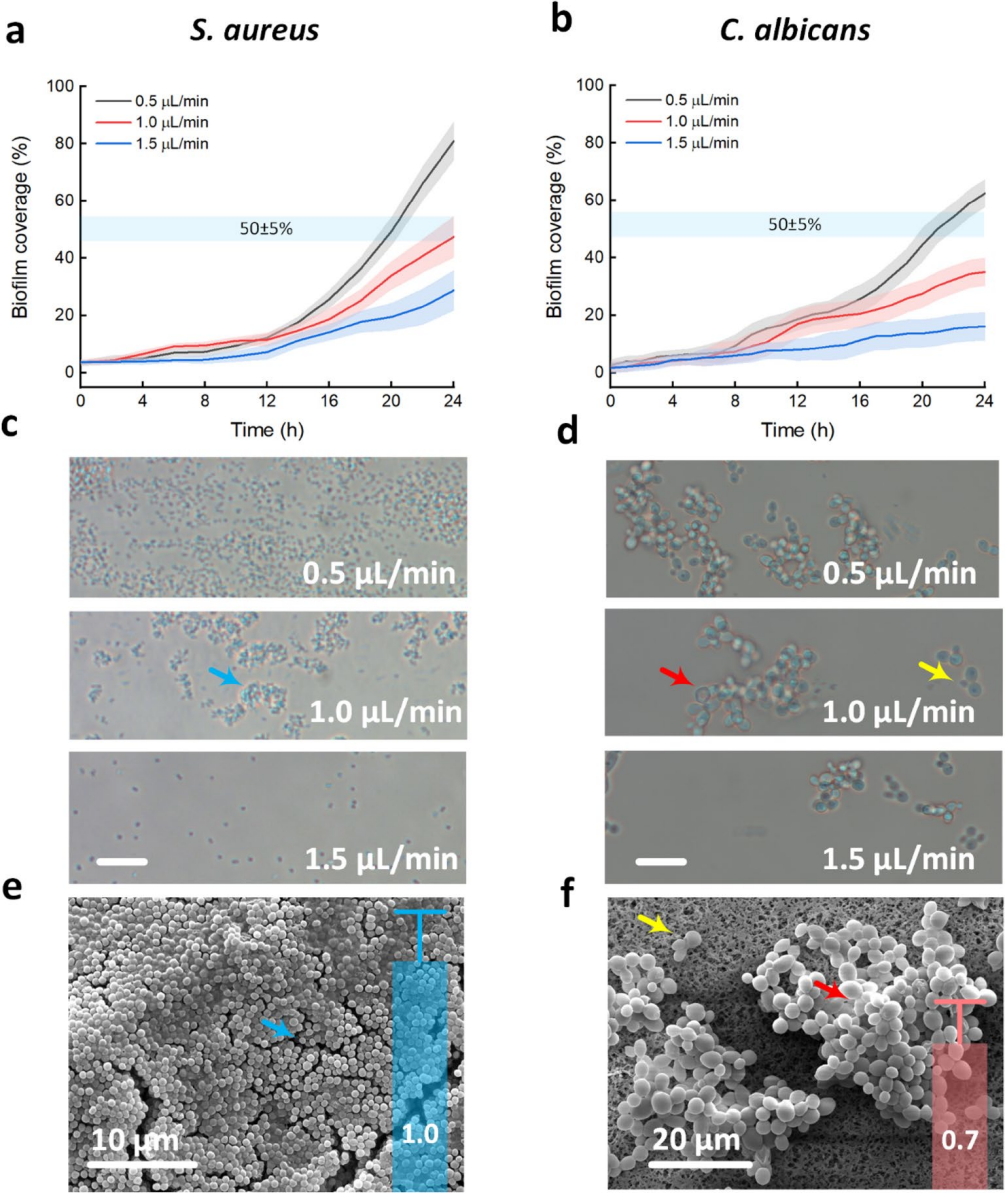
Comparison of biofilm coverages of mono-species biofilm formation under various flow rates (0.5, 1.0, and 1.5 µL/min) in microfluidic channels with static conditions grown on well plates after 24 h of culture. (**a**,**c**) *S. aureus* biofilm under hydrodynamic conditions. (**b**,**d**) *C. albicans* biofilm under hydrodynamic conditions. (**e**,**f**) Single-species biofilms (*S. aureus* vs. *C. albicans*) under static conditions (blue arrows = extracellular matrix; red arrows = pseudohyphae groups; yellow arrows = budding formation). The cyan lines represent the predetermined biofilm coverage (~ 50%) in the FOV on microchannels. Blue and red bar graphs in (**e**,**f**) show *S. aureus* and *C. albicans* biofilm growths in vitro on 96-well plates, respectively, measured by the optical density at 570 nm.

Figure 3 compares the biofilm coverages of mono-species biofilm formation in microfluidic channels at various flow rates with the biofilm grown under static conditions in microtiter plates. During the experiment, the frequency of *S. aureus* cell adhesion was higher than that of *C. albicans* cells. The microorganisms attached not only to the floor of the channels, but also to one another, forming very large aggregates. Furthermore, streamer formation was observed after inoculum injection into the microfluidic channels. The streamer regularly cleaned away the newly attached microorganisms on the glass surface. As a result, in this study, we integrated a herringbone mixer into the microfluidic channel. The flow of injected microorganisms was thoroughly mixed before passing through the observed channels. Furthermore, the channel was constructed in a conical shape at the inlets to analyze the biofilm formation at different flow rates in one channel (Fig. 1b). The microbial density decreased as the distance from the inlets increased owing to the increase in the flow velocity. Three channels were pumped at three distinct flow rates during a certain period of time. At the time of adhesion and proliferation, both microorganisms might experience high flow rates (flow rate = 1.5 µL/min; flow velocity = 25 mm/min). As the shear rate was 50 s^−1^ and the Reynolds number was 5.1 (*Re* < 100), the medium flow was considered laminar.

After 12 h of 1.5-µL/mm culture, *S. aureus* covered only 7% of the field of view (FOV), while *C. albicans* progressively attached and expanded to cover 16% of the FOV. On the other hand, *S. aureus* bacteria were enabled after 12 h of attachment and swiftly matured into significant biofilm after 24 h (biofilm coverage of 28.7%). As shown in Fig. 3c,d (bottom), both *S. aureus* and *C. albicans* scarcely produced dense biofilms. However, at 0.5 µL/mm, the almost non-adherent *S. aureus* only vibrated about themselves along the sidewalls and planktonic bacteria slowly flowed through the microfluidic channels. As a result of the flow of non-adherent planktonic *S. aureus*, single-cell movement was seldom detected. However, after 24 h of culture, we observed an overgrown biofilm containing planktonic bacteria (80.2% of the FOV). Similarly, the *C. albicans* biofilm developed modestly for 16 h before quickly proliferating over an additional 8 h (62.5% of the FOV; Fig. 3d; top). 1.0 µL/min was then used to grow both *S. aureus* and *C. albicans* biofilms, with the microbial biofilm covering approximately 50% of the FOV. Only attached microorganisms were visible under this hydrodynamic condition, while non-adherent microorganisms were eliminated by medium movement.

*S. aureus* and *C. albicans* produced biofilms with coverages of 49.5% and 35.1%, respectively. Figure 3c,d show the qualitative observations (middle). The extracellular matrix (ECM) of the *S. aureus* biofilms varied, whereas the *C. albicans* biofilms had the appearance of yeast, budding development, and apparent pseudohyphae. Under static conditions, the morphological types of the two microbial biofilms were equivalent to the low flow rate of dynamic conditions (0.5 µL/min). The *S. aureus* biofilm had a more spreading biofilm architecture, whereas the *C. albicans* biofilm had very large local aggregates (Fig. 3e,f). In good agreement with the SEM images, the measured optical densities of the *S. aureus* and *C. albicans* biofilms grown on 96-well plates were 1.0 and 0.7, respectively.

### Monitoring of single-cell tracking at the initial stage of biofilm formation

**Figure 4:**
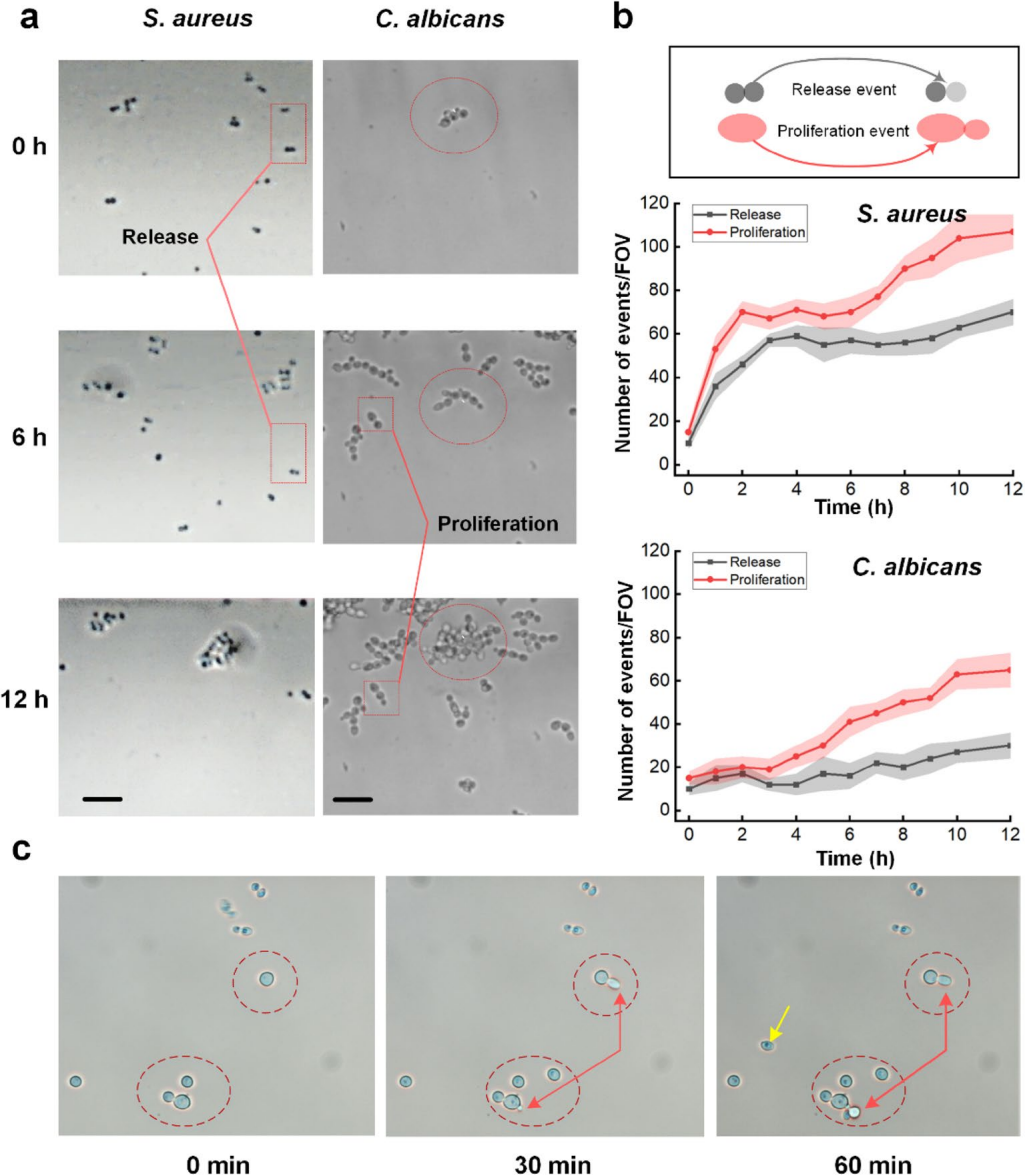
Single-cell tracking analysis of the early biofilm formation: (**a**) time-lapse microscopy images (left = *S. aureus*; right = *C. albicans*), (**b**) number of events during 12 h of culture (top = visualization of release and proliferation events; middle = number of events/FOV from *S. aureus* growth; bottom = number of events/FOV from *C. albicans* growth), and (**c**) budding formation of *C. albicans* cells at 0, 30, and 60 min (scale bar = 20 µm). The yellow arrow indicates the nucleus inside *C. albicans* cells.

Figure 4 shows a single-cell tracking investigation of the early biofilm development using time-lapse microscopy images of *S. aureus* and *C. albicans*. A proliferation event was defined as the creation of new microbes, whereas a release event was defined as the separation of a microbial from its mother cells or escape from the former location. We were able to analyze the biofilm development of various microorganisms under similar hydrodynamic and environmental conditions in one experiment by designing three separate channels on a chip. *C. albicans* colonized very quickly on the floor surface (Fig. 4a; left). After 12 h of incubation, a mature *C. albican*s biofilm was observed. On the other hand, *S. aureus* was attached, multiplied, and spread across the surroundings. Furthermore, the number of *S. aureus* cells adhering to the glass floor was larger than that of *C. albicans* cells. After 4 h of culture, we observed progressive growth and absence of discharge of *C. albicans* cells (Fig. 4b; bottom). After 30 min of adherence, a new budding development was observed. After that time, *C. albicans* cells began to release modestly. However, the number of observed multiplication events in *C. albicans* incubation was larger than the number of release cases. As a consequence, after 12 h of incubation, the biofilm grew gradually and matured. Despite the high number of release events, *S. aureus* exhibited a fast adhesion after 2 h of incubation (Fig. 4b; top). *S. aureus* bacteria were actively dividing at the start of the incubation. On the floor, there were more *S. aureus* cells than *C. albicans*. Despite this behavior, *S. aureus* continued to release and proliferate between 3 and 6 h, explaining the lag in bacterial colonization by *S. aureus*. After 6 h of culture, *S. aureus* cells multiplied significantly and produced a strong biofilm after 12 h of culture injection. These events were not observed in the *C. albicans* cells. Figure 4c shows the reproduction of *C. albicans* cells. A budding pattern was initially formed after approximately 30 min of injection, and the mother nucleus inside the fungus was divided into two parts. The daughter nucleus then moved to the budding pattern. Eventually, the daughter yeast separated from the mother and induced a budding scar on the cell surface.

### Monitoring of dual-species biofilm formation

**Figure 5:**
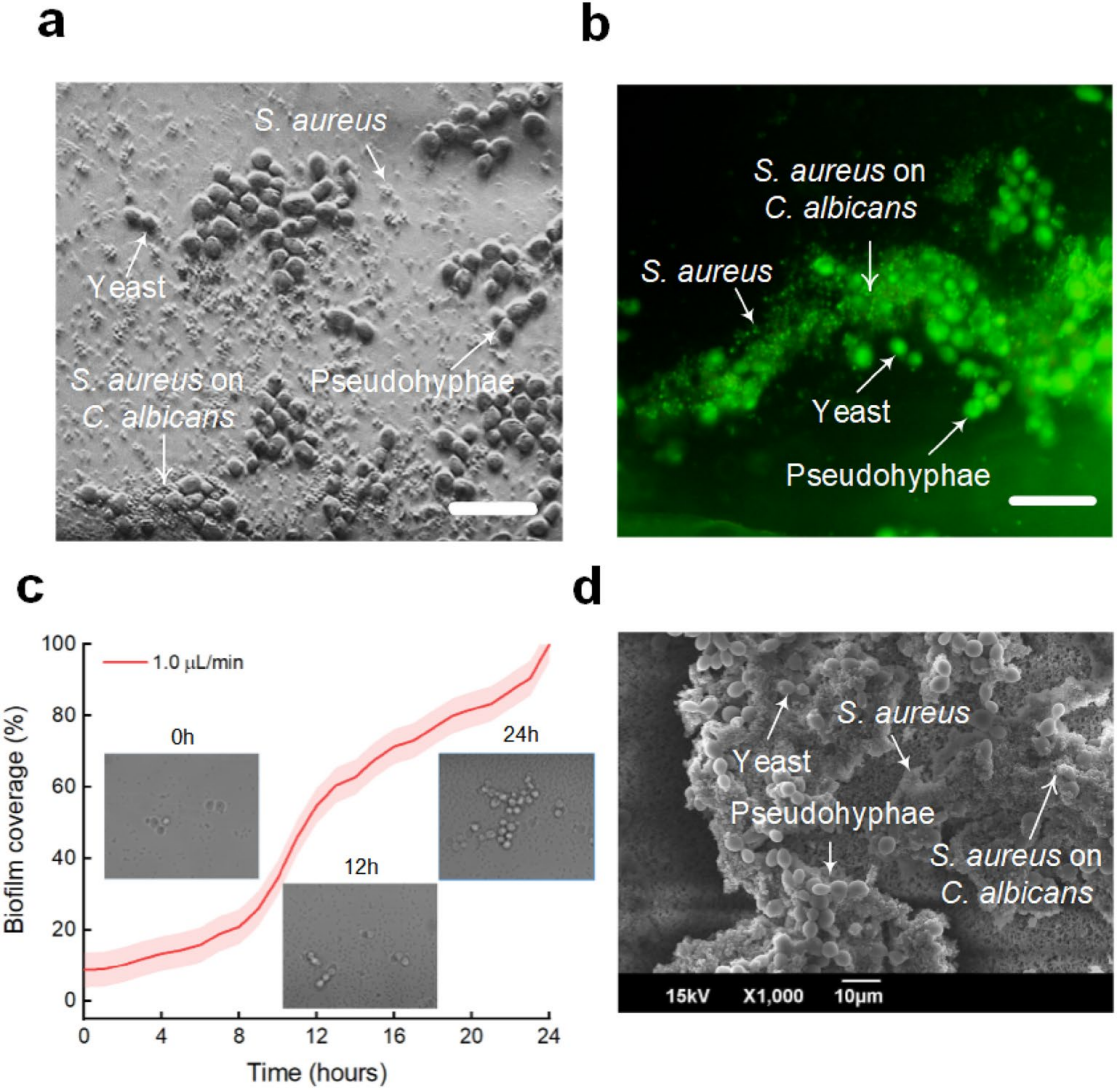
Biofilm coverage of dual-species biofilm formation (*S. aureus* + *C. albicans*): (**a**) bright-field image, (**b**) fluorescent image with SYTO-9 dye, (**c**) temporal development of biofilm coverage under a flow rate of 1 μL/min, and (**d**) SEM image under static conditions (scale bar = 20 μm).

Figure 5 illustrates the spatial morphology and physical interactions of dual-species biofilm formation by *S. aureus* and *C. albicans*. The bright-field image in Fig. 5a shows that *S. aureus* developed colonies not only on the floor surface but also on the *C. albicans* cell surfaces. Under hydrodynamic conditions, *S. aureus* bacteria were affected by the fluid flow, and non-adherent bacteria were flushed out towards the outlets. However, the *C. albicans* cell (yeast and pseudohyphal forms) surface acts as a scaffold, promoting the adherence of *S. aureus* cells. Figure 5a also shows several forms of *C. albicans* pathogens, such as yeast, budding form, and pseudohyphae groups, which result in increased resistance to antifungal germicides. In general, the dual biofilms exhibited an evolutive behaviour over time in terms of the number of cells, metabolic activity, and total biomass. The fluorescent image in Fig. 5b confirms the architecture of the dual biofilm. Two cells interacted with each other to form a condensed biofilm. *S. aureus* followed the *C. albicans* formation; therefore, a very small *S. aureus* biofilm was observed far from the *C. albicans* locations. Figure 5c shows the tendency of dual biofilm growth over 24 h of incubation. Within 4 h of the initial incubation, the numbers of releases and proliferation in the two pathogens were identical. Thus, the biofilm coverage was approximately 20% of the FOV. The dual-species biofilm was significantly developed from 6 to 12 h. However, 12 h later, the mixed biofilm formation seems to have slowly developed to cover 96.5% of the FOV after 24 h of incubation due to the stationary phase. When the objective of the inverted microscope was moved up and down, a thicker biofilm was clearly visible in the direction perpendicular to the floor surface. For comparison to the static conditions, the dual-species biofilm was also incubated on 96-well microtiter plates. In contrast, the two microorganisms evenly adhered and proliferated over the incubated surface (Fig. 5d). Notably, *C. albicans* pathogens grew in different phases, including yeast, budding formation, and pseudohyphae.

### Laser-light-mediated eradication of mature biofilms

**Figure 6:**
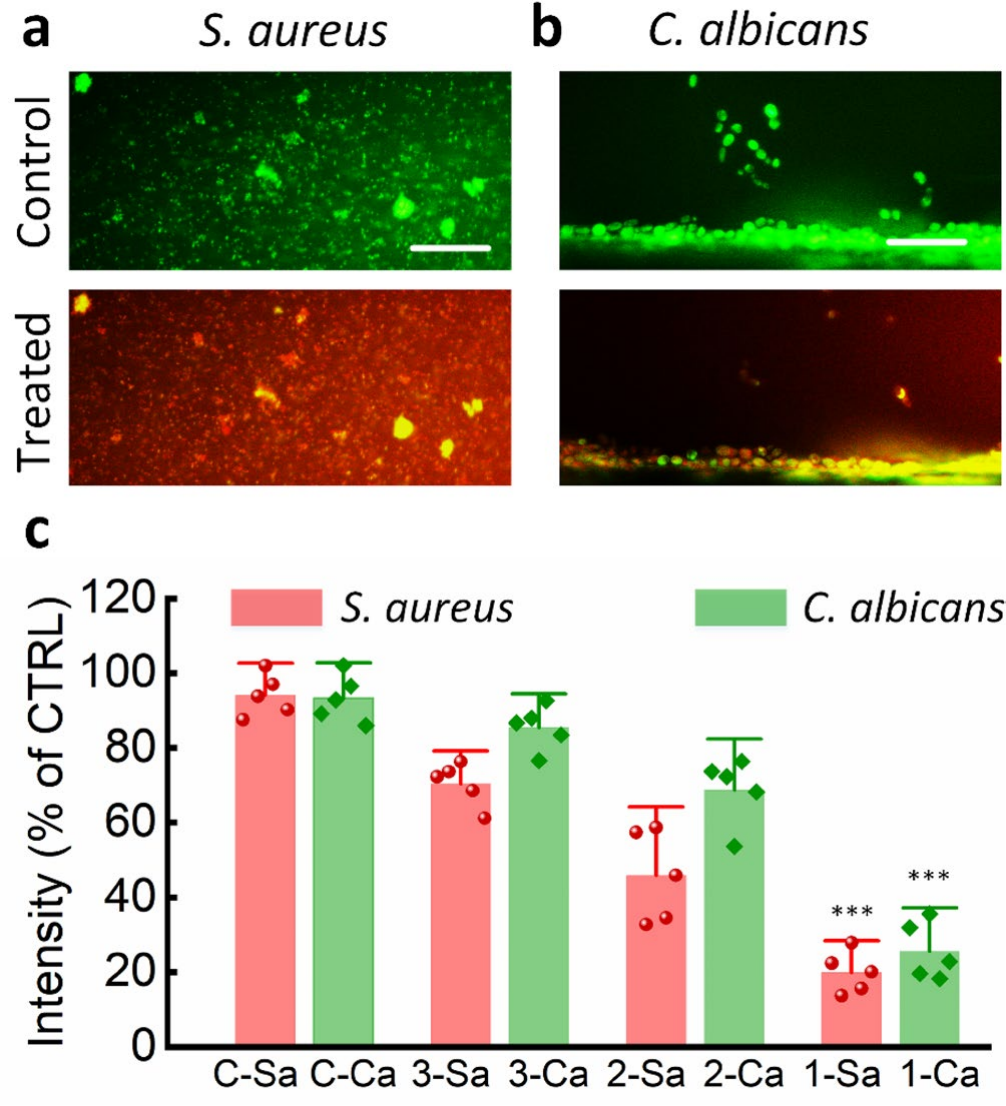
Laser treatment of the mono-species biofilm of *S. aureus* and *C. albicans* for 90 min at various irradiances: (**a**) untreated control (top) and treated samples (bottom) of the *S. aureus* biofilm, (**b**) untreated control (top) and treated samples (bottom) of *C. albicans* biofilm grown on channel 1, and (**c**) comparison of measured fluorescence intensities (fluences on channels 1, 2, and 3 = 1080, 540, and 270 J/cm^2^, respectively; scale bar = 20 μm; *C-Sa* untreated control samples of *S. aureus* biofilm, *1-Sa, 2-Sa, 3-Sa* treated samples of *S. aureus* on channels 1, 2, and 3, respectively, *C-Ca* untreated control samples of *C. albicans* biofilm, *1-Ca, 2-Ca, 3-Ca* treated samples of *C. albicans* on channels 1, 2, and 3, respectively; ***p < 0.01 vs. untreated control; *N* = 5).

Figure 6 shows the optimal laser dosage for successful biofilm eradication. The separate biofilm on each channel was irradiated by the predetermined optical energy because of the advancement of employing three channels in one chip and the integration of the optical system supply with three optical pathways (50%, 25%, and 12.5% of the output laser power). After 90 min of laser light irradiation, the vitalities of the *S. aureus* biofilms were 71.1%, 46.5%, and 19.8%, respectively, compared to untreated controls (Fig. 6c; *p* < 0.01).

**Figure 7:**
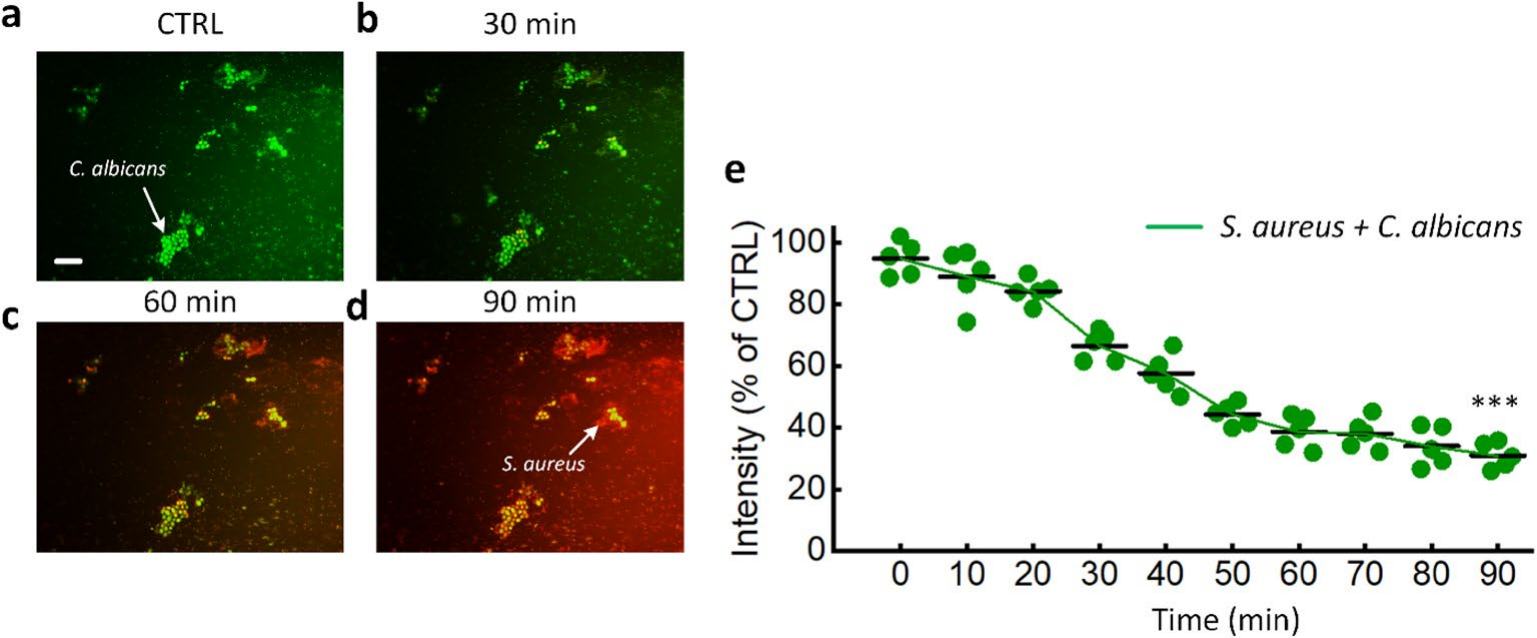
Laser treatment of the dual-species biofilm (*S. aureus* + *C. albicans*) under a flow rate of 1 µL/min: (**a**–**d**) time-lapse images of the samples after treatments for 0, 30, 60, and 90 min, respectively, and (**e**) green intensities measured from the untreated control (0 min) and treated samples (CTRL: untreated control; scale bar = 20 μm; ***p < 0.01 vs. untreated control; *N* = 5).

On the other hand, *C. albicans* biofilms were more resistant to laser light, with cell viabilities of 86.2%, 69.7%, and 26.4%, respectively, compared to untreated controls (Fig. 6c; *p* < 0.01). The maximum laser light irradiance for two separate biofilm treatments was then chosen for comparison to the cleaning effectiveness of the dual-species biofilm. Figure 7 shows the efficacy of laser therapy of the dual-species biofilms of *S. aureus* and *C. albicans* at a flow rate of 1 µL/min. The untreated control sample was completely green, which suggests that all cells were alive. On the other hand, the green intensity steadily diminished with time (Fig. 7a–d). The cell viability was determined to be 31.5% after 90 min of laser light exposure, compared to untreated controls (Fig. 7e; *p* < 0.01).

## Discussion

This study is significant because of the real-time tracking of the formation of mono- and dual-species biofilms of *S. aureu*s and *C. albicans* at a single-cell level, followed by the development of an antimicrobial therapy approach. The bacterial adherence was spatially controlled and homogenous on the microchannel floor. In this study, we designed and developed a microfluidic system for real-time studies on bacterial/fungal species adherence on surfaces and biofilm formation in a microchannel under well-controlled conditions. Tehranirokh et al. reported that the previously described microchannel size provided fine control of the hydrodynamic environments to study cell–cell and cell–ECM interactions^38^. Moreover, the current research involved culturing, allowing to establish the biofilms of fungal and bacterial cells either individually or in a mixed form using their respective growth media or equally mixed culture media. Thus, the cell culture of the two microbial species must be mixed completely before being inoculated into the observed channels on the microfluidic chip. Transverse flows were employed in this passive herringbone mixing approach to produce microvortices within microfluidic channels^38^. The purpose of using this innovative mixing technique was to control the concentrations of the individual cell culture at the initial mixing phase and investigate the entire interaction process during cross-kingdom biofilm formation. As a result, all microorganisms and growth media were thoroughly mixed during the establishment of both mono- and dual-species biofilms. Furthermore, three distinct phenotypic architectures of bacterial, fungal, and their mixed biofilms were simultaneously analyzed in one chip because of the three independent channel designs of the microfluidic device. As a result, the experiment can be completed three times faster than that with a single channel on one chip. The proposed setup originated from the lengthy time of conventional biofilm culture (culturing time > 24 h)^39^.

Using a well-established microfluidic device, the current findings showed that more adherent fungal cells in the injection phase did not result in larger surface colonization and biofilm development in comparison to the bacterial biofilm. This was attributed to a higher proclivity for bacteria to grow in a short period of incubation. In this investigation, the microbes remained adhered to the channel surface for 2 h, which was consistent with the previous studies^40^. Wang et al. confirmed that bacteria attached but did not proliferate, as single *E. coli* bacteria were discovered on the surfaces during the first 2 h. This phenomenon is known as the lag phase of the bacterial growth curve^40^. The bacterial adhesion and biofilm development are affected by experimental conditions (e.g., growth media and material surface characteristics) and types of bacterial species^41,42^.

However, in this study, at the flow rate of 0.5 µL/mm in the channel, almost non-adherent (planktonic) *S. aureus* cells vibrated along the sidewalls and slowly flowed through the microfluidic channels. Because of the random movement of planktonic bacterial cells, it is challenging to record images, which finally resulted in an erroneous coverage estimation of the simple biofilm. In vitro studies under static conditions also suffered from planktonic cells, which affect the quantification and further processing of the biofilm^43^. Thus, with the assistance of the newly developed microfluidic device, the hydrodynamic conditions were well controlled for single-cell investigation. As a result, the microfluidic technology developed in this study enabled automated single-cell tracking and the impact of hydrodynamic conditions during growth and biofilm formation in the fine channels. The microfluidic device was also integrated with laser irradiation, which helped in the real-time treatment of microbial cells at different flow rates. *S. aureus* bacterial cells proliferated and diffused faster than *C. albicans* cells, resulting in a more spreading mature biofilm architecture of *S. aureus*. The budding formation in *C. albicans* cells was apparently observed with the migration of the nucleus from the “mother” cells to “daughter” cells. After approximately 30 min of injection, a budding pattern emerged, and the mother nucleus inside the fungal cells was split into two parts. The daughter nuclei were then transferred to buds. Eventually, the daughter yeast splits from the mother and forms a budding scar on the cell surface^44^. Furthermore, *S. aureus* bacteria adhered to the channel floor and surface of *C. albicans* fungal cells to form a cross-kingdom biofilm. Reports showed that the *C. albicans* cell (yeast and pseudohyphal forms) surface acted as a scaffold to promote the adherence of *S. aureus* to the *C. albicans* cells in the process of cross-kingdom biofilm formation^18,45^. Thus, we observed a collective development of the mixed biofilm with a FOV coverage of 96.5% for 24 h of inoculation under a flow rate of 1 µL/min, which was almost twice faster compared to the *S. aureus* biofilm and three times faster compared to the *C. albicans* biofilm formation.

According to the proposed model, a microfluidic platform is appropriate for the analysis of the effect of growth factors, such as shear stress, on the above phenomena on various microbial species and platform stability and adaptability for testing novel antimicrobial agents. For example, compared to traditional antibiotic susceptibility testing, their effectiveness on surface-adherent bacteria may be assessed in a more therapeutically relevant environment. Furthermore, real-time monitoring of the antimicrobial activity of laser treatment at a wavelength of 405 nm at the single-cell level would provide a better understanding of its mode of action. In contrast to the original Gaussian and uncollimated beam profiles from the laser source, the laser beam on the microchannel surfaces was processed into flat-top and collimated forms (Fig. 2). Hence, the versatility of the optical system setup reduced the time and enabled a more precise measurement of biofilm eradication. After 90 min of laser light irradiation, the vitalities of the *S. aureus* and *C. albicans* biofilms were 19.8% and 26.4%, respectively, as evidenced by measuring the intensity (Fig. 6c; *p* < 0.01). In contrast, Peters et al. demonstrated that incubating *C. albicans* in 30% ethanol (EtOH) for 4 h was sufficient to kill and limit the growth, whereas 50% EtOH was required to block the *S. aureus* regrowth^46^. In other words, the *C. albicans* biofilms were more susceptible to EtOH than the *S. aureus* biofilms. In the case of the mixed biofilm, the cell viability was determined to be 31.5% after 90 min of laser light exposure (Fig. 7e), which indicates that the mixed biofilm was more resistant to laser irradiation than the mono-species biofilms. Lara et al. also reported that mixed bacterial-fungal biofilm infections are more difficult to treat than mono-species biofilm infections due to fungal ECM protection, which suggests a higher mortality rate and healthcare costs^47^. According to Zago et al., the coexistence of *S. aureus* and *C. albicans* in the biofilm developmental mode had an apparent collective impact, with bacterial cells preferentially associated with hyphal forms of the fungal cells^48^. Lin et al. proposed a plausible mechanism for the mixed biofilm by assuming planktonic forms of the two bacteria that might interact while are in suspension^49^. Coaggregation may help *C. albicans* acquire bulk, allowing it to sink to the channel floor^49^. This affinity of *S. aureus* for *C. albicans* hyphae might have a critical role in the enhanced virulence of infection maintained by this fungal-bacterial mixed biofilm^50^.

This study has an inherent constraint due to the use of only two-dimensional images with an inverted microscope, which might be overcome by employing confocal laser scanning microscopy with *z*-axis imaging^51^. Further studies will be needed to obtain high-resolution images of the polymicrobial interaction using a modified microbial strain with a fluorescent protein construct. Moreover, as the proposed platform enables the study of a wide range of microorganisms and growth conditions, studies on the formation of mono- and dual-species biofilms grown in a modified synthetic medium that mimics biological fluids and secretion will be carried out in the future. Finally, further research will continue to investigate the molecular mechanism of dual-species interactions in terms of real-time chemical (e.g., pH and O_2_) measurement, determination of EPS component distribution, measurement of microbial secondary metabolites, nutrient depletion, and morphological changes by using advanced microsensor, microscopy, metabolomics, and proteomics^52–57^. Furthermore, gene expression associated with biofilm and virulence will be investigated to examine the molecular mechanism^58,59^. The engineered microbial strain with the fluorescent protein construct will be used to monitor the high-resolution imaging of the polymicrobial interaction.

## Conclusion

This study shows an innovative microfluidic platform to monitor both formation and eradication of mono- and dual-species biofilms of bacterial (*S. aureus*) and fungal (*C. albicans*) pathogens in real-time. In contrast to static conditions, the physical interaction between *S. aureus* and *C. albicans* to form a dual-species biofilm was investigated under various hydrodynamic conditions. The proposed method can visualize the antimicrobial efficacy of the 405-nm laser light on the established mature mono- and dual-species biofilms of *S. aureus* and *C. albicans*. Further studies will analyze the effect of the microfluidic environment on the secretory molecules produced by bacterial or fungal species during biofilm development. The developed platform will be used to study antimicrobial therapy in situ against surface-associated biofilm-forming microbial pathogens.

## Materials and methods

### Microorganism samples

The bacterial strain *S. aureus* (KCTC 1916) was obtained from the Korean Collection for Type Cultures (KCTC, Daejeon, Korea), while the fungal strain *C. albicans* (KCCM 11282) was purchased from the Korean Culture Centre of Microorganisms (KCCM; Seodaemun-gu, Seoul, South Korea). The growth media for cultivating *S. aureus* and *C. albicans* were tryptic soy broth (TSB; Difco Laboratory Inc., Detroit, MI, USA) and potato dextrose broth supplemented with 5% glucose, respectively. The microbial cultures were grown in their growth media for mono-species biofilm, whereas the mixed culture growth media (50% TSB and 50% PDB) was employed for mixed-biofilm growth^60^. These microorganisms were stored at − 70 °C in 20% glycerol. The temperature for seed culture preparation and sub-culturing of microorganisms on an agar plate was 37 °C.

### Microfluidic device

Supplementary Fig. S1 depicts the design of the microfluidic device and experimental setup for biofilm growth. Three independent channels on one platform (distance between two channels = 7 mm) were designed to simultaneously analyze the different hydrodynamic conditions (i.e., three flow rates). Each channel has two inlets for microorganisms and medium supply and one outlet for waste collection (see Supplementary Fig. S1a,b). Each observed channel had a width of 100 µm and a height of 180 µm (see Supplementary Fig. S1a). The customized polydimethylsiloxane microfluidic chip was designed using the SolidWorks program (Dassault Systèmes SolidWorks Co., France). A photoresist (PR) mould for the micromixer was fabricated by two-step photolithography using a negative PR (SU-2050, MicroChem Corp, USA). The PR for the first channel layer was spin-coated on a silicon wafer at 500 rpm for 15 s and 1500 rpm for 40 s. The same spin coating was repeated to define the second ridge layer after PR baking and ultraviolet exposure. These spinning conditions resulted in a channel height of 160–165 μm and ridge height of 55–80 μm. The chip was constructed on a 76 mm × 26 mm glass slide (see Supplementary Fig. S1c). At the initial injection stage of microorganisms and medium, a herringbone mixer was built on the microfluidic chip to mix the microorganisms and medium before they were inoculated into the observed channels^61^. Tygon tubes (Saint-Gobain Performance Plastics Inc., France) were used to link the microfluidic inlets to the infusion pumps (New Era Pump Systems, Inc., USA). To evaluate the mixing capability of the fabricated chip, two-colour pigments (blue = erioglaucine disodium salt; yellow = tartrazine) were deployed and evaluated by measuring the grey value.

### Biofilm formation in microfluidic channels

**Figure 8:**
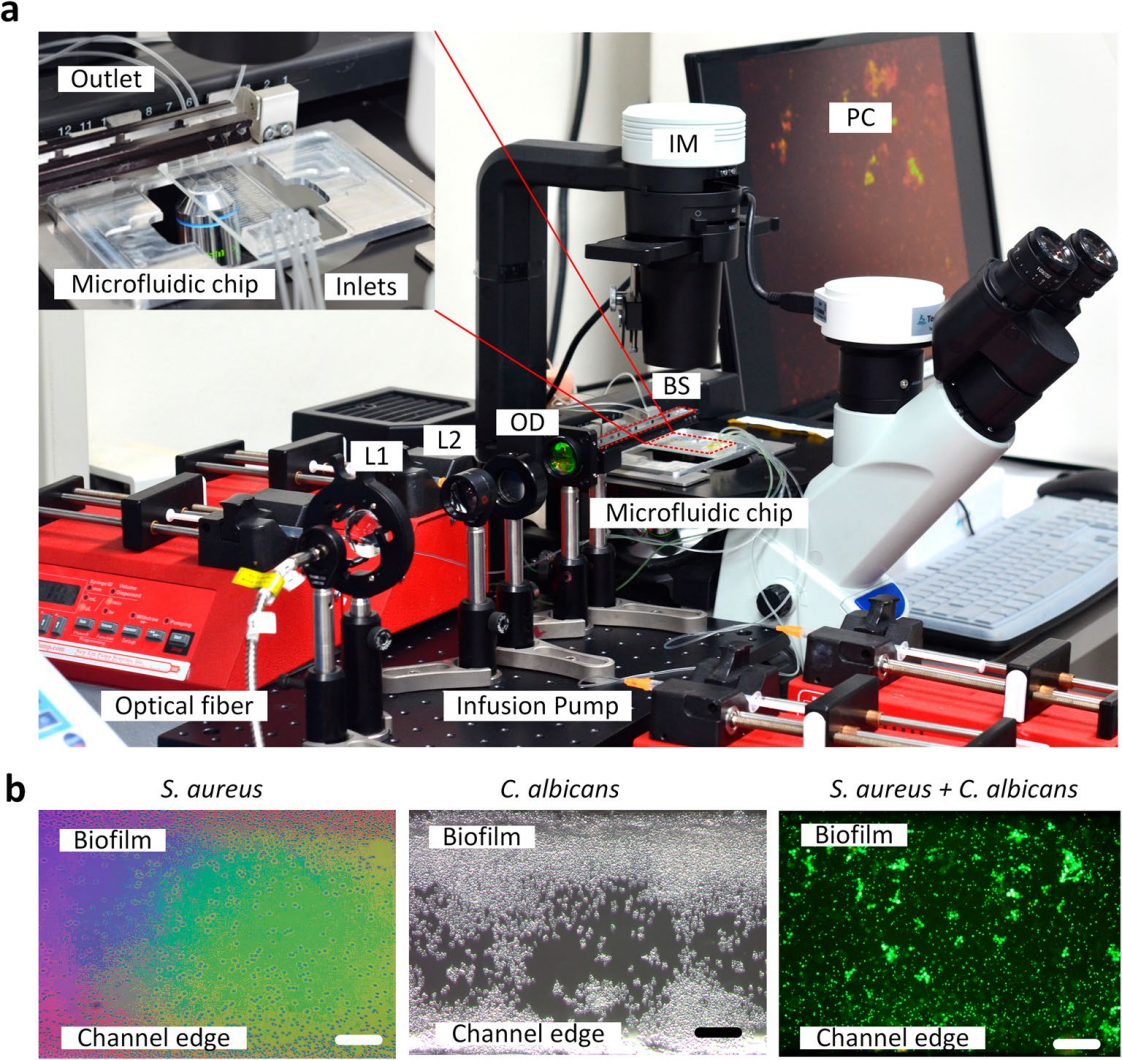
Experimental setup of the opto-microfluidic platform for biofilm culture and treatment: (**a**) experimental setup and (**b**) *S. aureus* biofilm (left), *C. albicans* biofilm (middle), and dual-species biofilm (*S. aureus* + *C. albicans*; right) (*IM* inverted microscope, *L1–L2* lenses, *OD* optical diffuser, *BS* beam splitter, *PC* personal computer; scale bar = 25 µm).

The biofilm formation (*S. aureus* and *C. albicans*) in the microfluidic channel was carried out according to a reported procedure with a slight modification^36^. The overnight-grown *S. aureus* (grown in TSB) and *C. albicans* (grown in potato dextrose broth with 5% glucose) were diluted (1:100, equivalent to an *OD*_600_ value of 0.05) in their respective growth media. To avoid contamination, the microfluidic platform was washed twice with 70% ethyl alcohol and washed again using a sterile growth medium. To establish a mono-species biofilm, the diluted microorganism culture prepared in their respective growth media was fully injected into the microfluidic channel and allowed to rest for 2 h to attach to the inner surface of the channel (Fig. 8a). On the other hand, for the establishment of a dual-species biofilm of *S. aureus* and *C. albicans*, an equal volume of the diluted microorganism cell culture (prepared in their respective growth media) was pumped into the microfluidic channel using infusion pumps (NE-1000, New Era Pump Systems, Inc., NY, USA)*.* The outlets were manually locked to completely remove the residual air bubbles from the channels because of the accumulated high pressure. At the inlets of each channel, two air traps were deployed to avoid air bubble formation in the channels when the fresh culture/medium was changed during the experiments. The hydrodynamic conditions were controlled using infusion pumps for a further 24 h of incubation (Fig. 8a). Based on the previous report, the three flow rates were set to 0.5, 1.0, and 1.5 µL/min (flow velocity = 27.8, 55.6, and 83.4 mm/min, respectively)^62,63^. An inverted microscope (CKX-53, Olympus, USA) was used to visually monitor the real-time processes of microbial adherence, biofilm formation, and optical treatment. The biofilm formation was analyzed and recorded every 2 h. All experiments were carried out at 25 °C (room temperature). Figure 8b shows *S. aureus* bacterial (left), *C. albicans* (middle), and mixed (right) biofilms formed on the current microfluidic channels.

### Shear stress and Reynolds number determination

The Reynolds number (*Re*) is a vital dimensionless parameter in viscous flows, particularly in the presence of various length scales. To design a well-characterised microfluidic platform, the flow is usually recommended to have *Re* < 100 to ensure laminar streams in the channels. *Re* can be expressed by^64^

1 $${\text{Re}} = \rho D\upsilon /\mu ,$$

where $\rho$, *D*, and *μ* are the density of the medium (1000 kg/m^3^), the hydraulic diameter of the channel (100 μm), the flow velocity of the medium on the channels (27.8, 55.6, and 83.4 mm/min for flow rates of 0.5, 1.0, and 1.5 µL/min, respectively), and dynamic viscosity of the medium (0.89 mPa)^65^, respectively.

The shear stress on the microchannel floor was calculated by^64^

2 $$\gamma = 6Q/wh^{2} ,$$

where *Q*, *w*, and *h* are the volumetric flow rate (μm^3^/s), channel width (180 μm), and channel height (100 μm), respectively.

### Optical setup

A free online software 3DOPTIX (3DOptix Co., Tel Aviv, Israel) was used to perform a ray-tracing model. A 405-nm light source (CNI Co., Changchun, China) was used to analyze the antimicrobial effect of laser light on mature mono- and dual-species biofilms (Fig. 8). Two lenses were used to collimate the incident laser beam (an aspheric lens with an effective focal length of 25 mm and a plan-convex lens with an EFL of 6 mm; Edmund Optics Co., City, State, USA). An optical diffuser (Edmund Optics Co., City, State, USA) was used to reshape the collimated Gaussian beam to apply spatially uniform emissions on microfluidic channels. This approach enabled the microfluidic platform to move freely while maintaining a consistent intensity of the laser light on the channels. To determine the effective dose of the 405-nm laser light on the microbial biofilms, three beam splitters (ratio = 50:50; Edmund Optics Co., USA) were used to deliver on the microfluidic channels. The delivered laser light onto the observed channels was applied with decreasing doses, such as 50%, 25%, and 12.5%, for 90 min with laser irradiances of 0.2, 0.1, and 0.05 W/cm^2^ and laser fluences of 1080, 540, and 270 J/cm^2^, respectively. After every 10 min, the effect of the laser treatment against mono- and dual-species biofilms was recorded.

### Biofilm characterization

Microfluidic channels were observed in real-time using an inverted microscope (CKX-53, Olympus, USA) with magnifications of 20 × and 40 × (bright-field and phase contrast) and a digital camera (Nikon D3500 DSLR, Nikon Inc., USA). To determine the biofilm coverage, the established visible biofilms on the channel floor of the microfluidic platform (floor area ~ 130 × 100 µm^2^) were captured by the microscope at three random sites (for each measurement, *N* = 3). All the acquired microscopic images were then converted into 8-bit formats and processed by using the software Fiji (National Institutes of Health, Bethesda, MD). The biofilm coverage was then presented as the percentage (%) of the measured biofilm area to the captured area. The TrackMate plugin was used to track the single-cell movement of the early biofilm formation for 12 h on a subset of the FOV^66^. Fluorescence imaging was performed with SYTO-9 and propidium iodide dyes to visualize live (stained with green colour) and dead (stained with red colour) cells^67^.

To compare biofilm formation in the microfluidic device to static conditions (without shaking at 25 °C), the overnight-grown cell cultures of *S. aureus* and *C. albicans* were diluted in their respective growth media with a normalized OD_600_ of 0.05. For the mono-species biofilm assays, the diluted cell culture in the growth medium was inoculated into a 96-well microtiter plate and incubated for 24 h. Similarly, for the dual-species biofilm assays, equal volumes of diluted (*OD*_600_ = 0.05) cultures of *S. aureus* and *C. albicans* were inoculated into the microplate and incubated for 24 h. After the free-floating cells were discarded, the surface-attached cells were washed with water and stained with 0.1% crystal violet. The stained cell culture was dissolved in 95% ethyl alcohol and quantified at 570 nm using a microplate reader (BioTek, Winooski, VT, USA).

Both mono- and dual-species biofilm architectures established under static conditions were visualized using SEM (TESCAN; Vega II LSU, Czech). Mono- and dual-species biofilms were grown on the surface of a nylon membrane (0.5 × 0.5 cm) and placed in a 24-well microplate. After 24 h of incubation at 25 °C, the cell culture was directly fixed with formaldehyde and glutaraldehyde overnight at 4 °C. The fixed cells were washed three times with phosphate-buffered saline (pH = 7.4), followed by dehydration with increasing concentrations (50–100%) of ethyl alcohol. A freeze dryer (FD8518, ilShinBiobase Co. Ltd., Korea) was used to dry the membrane. The biofilm architecture of the nylon membrane was visualized using an electron microscope.

### Statistical analysis

The data are presented as mean ± standard deviation. Each group (both treated and untreated control samples) was measured five times (N = 5). Because of the small number of test samples and distribution-free data, the Mann–Whitney *U* test was used for a nonparametric statistical analysis to evaluate the differences among the groups^46^. A Bonferroni correction with an adjusted *p*-value was used as a post hoc test to minimize the chances of incorrectly rejecting the null hypothesis when each pair of the groups was compared^67,68^. An SPSS program (SPSS, Inc., Chicago, USA) was used to assist the calculation process, and *p* < 0.5 was considered statistically significant^69^.

## Supplementary Information

Supplementary Figure S1.

## Data Availability

The datasets generated during and/or analyzed during the current study are available from the corresponding authors on reasonable request.

## References

[1] Li X (2021). Saliva-derived microcosm biofilms grown on different oral surfaces in vitro. NPJ Biofilms Microbiomes.

[2] Mahnert A (2019). Man-made microbial resistances in built environments. Nat. Commun..

[3] Hibbing ME, Fuqua C, Parsek MR, Peterson SB (2010). Bacterial competition: Surviving and thriving in the microbial jungle. Nat. Rev. Microbiol..

[4] Peters BM, Jabra-Rizk MA, O'May GA, Costerton JW, Shirtliff ME (2012). Polymicrobial interactions: Impact on pathogenesis and human disease. Clin. Microbiol. Rev..

[5] Donlan RM (2002). Biofilms: Microbial life on surfaces. Emerg. Infect. Dis..

[6] Gebreyohannes G, Nyerere A, Bii C, Sbhatu DB (2019). Challenges of intervention, treatment, and antibiotic resistance of biofilm-forming microorganisms. Heliyon.

[7] Koo H, Allan RN, Howlin RP, Stoodley P, Hall-Stoodley L (2017). Targeting microbial biofilms: Current and prospective therapeutic strategies. Nat. Rev. Microbiol..

[8] Singh S, Singh SK, Chowdhury I, Singh R (2017). Understanding the mechanism of bacterial biofilms resistance to antimicrobial agents. Open Microbiol. J..

[9] Faix R, Kovarik S (1989). Polymicrobial sepsis among intensive care nursery infants. J. Perinatol..

[10] Mohan R (2015). A microfluidic approach to study the effect of bacterial interactions on antimicrobial susceptibility in polymicrobial cultures. RSC Adv..

[11] Brown SP, Hochberg ME, Grenfell BT (2002). Does multiple infection select for raised virulence?. Trends Microbiol..

[12] Rodrigues ME, Gomes F, Rodrigues CF (2020). *Candida* spp./bacteria mixed biofilms. J. Fungi.

[13] Shirtliff ME, Peters BM, Jabra-Rizk MA (2009). Cross-kingdom interactions: *Candida albicans* and bacteria. FEMS Microbiol. Lett..

[14] Carolus H, Van Dyck K, Van Dijck P (2019). *Candida albicans* and *Staphylococcus* species: A threatening twosome. Front. Microbiol..

[15] Khan F (2021). Mixed biofilms of pathogenic *Candida*-bacteria: Regulation mechanisms and treatment strategies. Crit. Rev. Microbiol..

[16] Oogai Y (2011). Expression of virulence factors by *Staphylococcus aureus* grown in serum. Appl. Environ. Microbiol..

[17] Khan F (2021). Suppression of hyphal formation and virulence of *Candida albicans* by natural and synthetic compounds. Biofouling.

[18] Peters BM (2012). *Staphylococcus aureus* adherence to *Candida albicans* hyphae is mediated by the hyphal adhesin Als3p. J. Microbiol..

[19] Harriott MM, Noverr MC (2009). *Candida albicans* and *Staphylococcus aureus* form polymicrobial biofilms: Effects on antimicrobial resistance. Antimicrob. Agents Chemother..

[20] Todd OA (2019). *Candida albicans* augments *Staphylococcus aureus* virulence by engaging the staphylococcal *agr* quorum sensing system. MBio.

[21] Peters BM, Noverr MC (2013). *Candida albicans-Staphylococcus aureus* polymicrobial peritonitis modulates host innate immunity. Infect. Immun..

[22] Todd OA, Peters BM (2019). *Candida albicans* and *Staphylococcus aureus* pathogenicity and polymicrobial interactions: Lessons beyond Koch’s postulates. J. Fungi.

[23] Krause J, Geginat G, Tammer I (2015). Prostaglandin E2 from *Candida albicans* stimulates the growth of *Staphylococcus aureus* in mixed biofilms. PLoS One.

[24] Rusconi R, Stocker R (2015). Microbes in flow. Curr. Opin. Microbiol..

[25] Kou S, Cheng D, Sun F, Hsing I-M (2016). Microfluidics and microbial engineering. Lab Chip.

[26] Kim J, Park H-D, Chung S (2012). Microfluidic approaches to bacterial biofilm formation. Molecules.

[27] Crabbé A (2008). Use of the rotating wall vessel technology to study the effect of shear stress on growth behaviour of *Pseudomonas aeruginosa* PA01. Environ. Microbiol..

[28] Pérez-Rodríguez S, García-Aznar JM, Gonzalo-Asensio J (2022). Microfluidic devices for studying bacterial taxis, drug testing and biofilm formation. Microb. Biotechnol..

[29] Straub H (2020). A microfluidic platform for in situ investigation of biofilm formation and its treatment under controlled conditions. J. Nanobiotechnol..

[30] Kim KP (2010). In situ monitoring of antibiotic susceptibility of bacterial biofilms in a microfluidic device. Lab Chip.

[31] Yawata Y (2010). Monitoring biofilm development in a microfluidic device using modified confocal reflection microscopy. J. Biosci..

[32] Holman H-YN (2009). Real-time chemical imaging of bacterial activity in biofilms using open-channel microfluidics and synchrotron FTIR spectromicroscopy. J. Anal. Chem..

[33] Tremblay YD, Vogeleer P, Jacques M, Harel J (2015). High-throughput microfluidic method to study biofilm formation and host–pathogen interactions in pathogenic *Escherichia*
*coli*. Appl. Environ. Microbiol..

[34] Nance WC (2013). A high-throughput microfluidic dental plaque biofilm system to visualize and quantify the effect of antimicrobials. J. Antimicrob. Chemother..

[35] Hansen MF, Torp AM, Madsen JS, Røder HL, Burmølle M (2019). Fluidic resistance control enables high-throughput establishment of mixed-species biofilms. Biotechniques.

[36] Kasetty S, Mould DL, Hogan DA, Nadell CD (2021). Both *Pseudomonas aeruginosa* and *Candida albicans* accumulate greater biomass in dual-species biofilms under flow. mSphere.

[37] Tran VN, Dasagrandhi C, Truong VG, Kim Y-M, Kang HW (2018). Antibacterial activity of *Staphylococcus aureus* biofilm under combined exposure of glutaraldehyde, near-infrared light, and 405-nm laser. PLoS One.

[38] Stott SL (2010). Isolation of circulating tumor cells using a microvortex-generating herringbone-chip. Proc. Natl. Acad. Sci. U.S.A..

[39] Khomtchouk K (2019). Quantitative assessment of bacterial growth phase utilizing flow cytometry. J. Microbiol. Methods.

[40] Wang L, Fan D, Chen W, Terentjev EM (2015). Bacterial growth, detachment and cell size control on polyethylene terephthalate surfaces. Sci. Rep..

[41] Petrova OE, Sauer K (2012). Sticky situations: Key components that control bacterial surface attachment. J. Bacteriol..

[42] Hancock V, Witsø IL, Klemm P (2011). Biofilm formation as a function of adhesin, growth medium, substratum and strain type. J. Med. Microbiol..

[43] Merritt JH, Kadouri DE, O'Toole GA (2011). Growing and analyzing static biofilms. Curr. Protoc. Microbiol..

[44] Kabir MA, Hussain MA, Ahmad Z (2012). *Candida albicans*: A model organism for studying fungal pathogens. Int. Sch. Res. Notices.

[45] Peters BM (2010). Microbial interactions and differential protein expression in *Staphylococcus aureus–Candida albicans* dual-species biofilms. FEMS Microbiol. Immunol..

[46] Peters BM, Ward RM, Rane HS, Lee SA, Noverr MC (2013). Efficacy of ethanol against *Candida albicans* and *Staphylococcus aureus* polymicrobial biofilms. Antimicrob. Agents Chemother..

[47] Lara HH, Lopez-Ribot JL (2020). Inhibition of mixed biofilms of *Candida albicans* and methicillin-resistant *Staphylococcus aureus* by positively charged silver nanoparticles and functionalized silicone elastomers. Pathogens.

[48] Zago CE (2015). Dynamics of biofilm formation and the interaction between *Candida albicans* and methicillin-susceptible (MSSA) and-resistant *Staphylococcus aureus* (MRSA). PLoS One.

[49] Lin YJ (2013). Interactions between *Candida albicans* and *Staphylococcus aureus* within mixed species biofilms. Bios.

[50] Tambone E (2021). Counter-acting *Candida albicans*–*Staphylococcus aureus* mixed biofilm on titanium implants using microbial biosurfactants. Polymers.

[51] Venkateswarlu K (2020). Three-dimensional imaging and quantification of real-time cytosolic calcium oscillations in microglial cells cultured on electrospun matrices using laser scanning confocal microscopy. Biotechnol. Bioeng..

[52] Schlafer S, Kamp A, Garcia JE (2018). A confocal microscopy based method to monitor extracellular pH in fungal biofilms. FEMS Yeast Res..

[53] von Ohle C (2010). Real-time microsensor measurement of local metabolic activities in ex vivo dental biofilms exposed to sucrose and treated with chlorhexidine. Appl. Environ. Microbiol..

[54] Pousti M, Zarabadi MP, Amirdehi MA, Paquet-Mercier F, Greener J (2019). Microfluidic bioanalytical flow cells for biofilm studies: A review. Analyst.

[55] Liu X (2011). Real-time mapping of a hydrogen peroxide concentration profile across a polymicrobial bacterial biofilm using scanning electrochemical microscopy. Proc. Natl. Acad. Sci. U.S.A..

[56] Dige I, Baelum V, Nyvad B, Schlafer S (2016). Monitoring of extracellular pH in young dental biofilms grown in vivo in the presence and absence of sucrose. J. Oral Microbiol..

[57] Choong FX (2021). A semi high-throughput method for real-time monitoring of curli producing *Salmonella* biofilms on air-solid interfaces. Biofilm.

[58] DelMain EA (2020). Stochastic expression of Sae-dependent virulence genes during *Staphylococcus aureus* biofilm development is dependent on SaeS. MBio.

[59] Short, B. *et al.* Investigating the transcriptome of *Candida albicans* in a dual-species *Staphylococcus**aureus* biofilm model. *Front. Cell. Infect. Microbiol.* 1142 (2021). 10.3389/fcimb.2021.791523 PMC8650683 34888261

[60] Lee J-H (2019). Inhibition of biofilm formation by *Candida albicans* and polymicrobial microorganisms by nepodin via hyphal-growth suppression. ACS Infect. Dis..

[61] Forbes TP, Kralj JG (2012). Engineering and analysis of surface interactions in a microfluidic herringbone micromixer. Lab Chip.

[62] Sakamoto C (2007). Rapid quantification of bacterial cells in potable water using a simplified microfluidic device. J. Microbiol. Methods.

[63] Richter L (2007). Development of a microfluidic biochip for online monitoring of fungal biofilm dynamics. Lab Chip.

[64] Young EW, Simmons CA (2010). Macro-and microscale fluid flow systems for endothelial cell biology. Lab Chip.

[65] Petrochenko PE (2018). Analytical considerations for measuring the globule size distribution of cyclosporine ophthalmic emulsions. Int. J. Pharm..

[66] Tinevez J-Y (2017). TrackMate: An open and extensible platform for single-particle tracking. Methods.

[67] Tran VN (2021). Opto-chemical treatment for enhanced high-level disinfection of mature bacterial biofilm in a Teflon-based endoscope model. Biomed. Opt. Express.

[68] Staffa SJ, Zurakowski D (2020). Strategies in adjusting for multiple comparisons: A primer for pediatric surgeons. J. Pediatr. Surg..

[69] Tran VN (2022). Collective bacterial disinfection by opto-chemical treatment on mature biofilm in clinical endoscope. J. Photochem. Photobiol. B Biol..

