# Supplementary material for: Real-time monitoring of mono- and dual-species biofilm formation and eradication using microfluidic platform
Source: Sci Rep. 2022 Jun 11;12:9678. doi: 10.1038/s41598-022-13699-9 (PMC9188611; doi:10.1038/s41598-022-13699-9)
Supplement: Supplementary file 1 — Supplementary Figure S1. [file 41598_2022_13699_MOESM1_ESM.docx]

**Supplementary Information**

**Supplementary Figure S1.** Design of microfluidic device: **(a)** computer-aided design (CAD) of microfluidic chip with three independent channels, **(b)** microfluidic device prototype on glass slide (76 mm × 26 mm), and **(c)** fabrication protocols of microfluidic device (B: Bacteria; Y: Yeast; M: Media; HM: herringbone mixer; OC: observed channel; PDMS: Polydimethylsiloxane). Note that solid red arrows indicate the direction of bacteria/yeast/media flow through the microchannels.

**
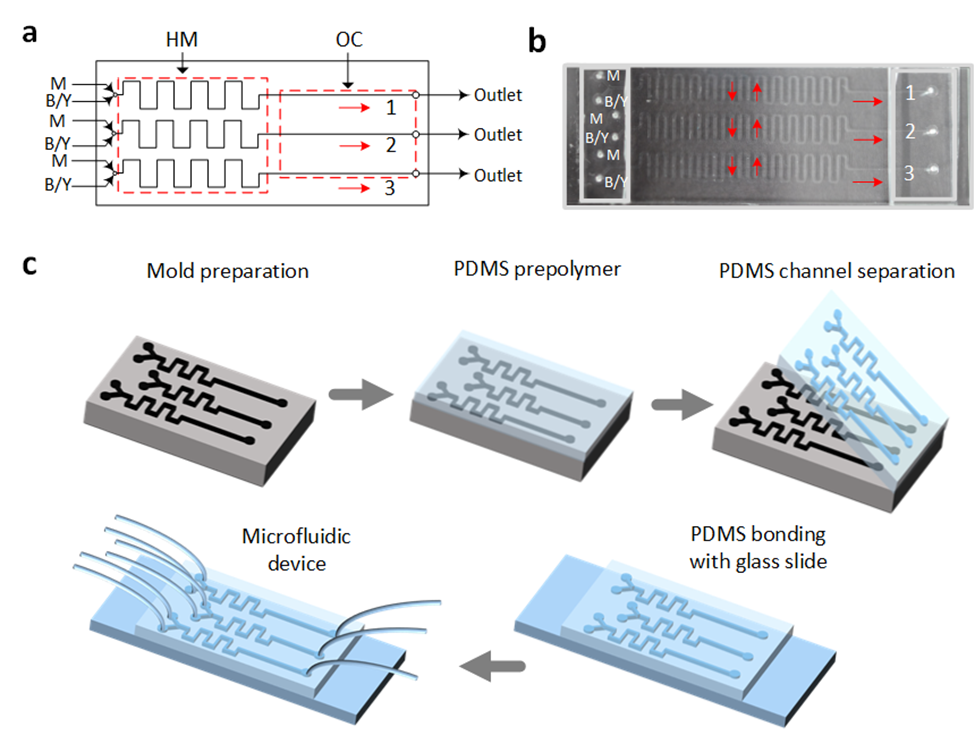
**
